# The Kansas City Homœopathic College*We publish this interesting letter of Dr. Waggoner with the desire of calling the attention of the profession to the new college that is now flourishing in this part of our country, and to commend it to their good-will.

**Published:** 1890-05

**Authors:** Annette H. Waggoner

**Affiliations:** Kansas City, Kansas


### THE KANSAS CITY HOMŒOPATHIC COLLEGE.*

KANSAS CITY, KANSAS, March 16th, 1890.

EDITORS OF THE HOMŒOPATHIC PHYSICIAN:—I am glad to have recalled to your mind some recollections of the two Kansas Cities. One of them, as is well known, is Kansas City, Missouri, the other is just across the boundary line—the Kaw River—in Kansas. I live in the latter city. Great changes have taken place since you were here. Even in the three years that I have been a resident the improvement is wonderful.

To my mind, the rapid building up of attractive homes in the suburbs is the most interesting. But it is of a different subject I wish to tell you.

Two and a half years ago a Homœopathic Medical School was organized in Kansas City, Missouri, the Faculty composed of several physicians of that side of the river and three from this, who gave their services and advanced their money "for the good of the cause."

Last Thursday evening I attended their second commencement, of which I inclose a programme.

The institution is now out of debt, with money in the treasury, and increasing facilities for instruction.

Of eighteen students six were graduates, of whom four were ladies.

The valedictory was far more interesting than those productions usually are, and the appearance and manner of the lady who read it added to the pleasing effect on the large and appreciative audience, warming the hearts of the women of our profession with gratified pride.

There are several allopathic schools in the city, but this is the first exclusively homœopathic institution.

Respectfully,

ANNETTE H. WAGGONER, M. D.

---

*We publish this interesting letter of Dr. Waggoner with the desire of calling the attention of the profession to the new college that is now flourishing in this part of our country, and to commend it to their good-will.

